# The effects of Rho-associated kinase inhibitor Y-27632 on primary human corneal endothelial cells propagated using a dual media approach

**DOI:** 10.1038/srep09167

**Published:** 2015-03-16

**Authors:** Gary S. L. Peh, Khadijah Adnan, Benjamin L. George, Heng-Pei Ang, Xin-Yi Seah, Donald T. Tan, Jodhbir S. Mehta

**Affiliations:** 1 Tissue Engineering and Stem Cell Group, Singapore Eye Research Institute, Singapore; 2 Duke-NUS Graduate Medical School, Singapore; 3 Singapore National Eye Centre, Singapore; 4 School of Material Science and Engineering, Nanyang Technological University, Singapore; 5 Yong Loo Lin School of Medicine, National University of Singapore, Singapore

## Abstract

The global shortage of donor corneas has garnered extensive interest in the development of graft alternatives suitable for endothelial keratoplasty using cultivated primary human corneal endothelial cells (CECs). We have recently described a dual media approach for the propagation of human CECs. In this work, we characterize the effects of a Rho-kinase inhibitor Y-27632 on the cultivation of CECs propagated using the dual media culture system. Seventy donor corneas deemed unsuitable for transplantation were procured for this study. We assessed the use of Y-27632 for its effect at each stage of the cell culture process, specifically for cell attachment, cell proliferation, and during both regular passaging and cryopreservation. Lastly, comparison of donor-matched CEC-cultures expanded with or without Y-27632 was also performed. Our results showed that Y-27632 significantly improved the attachment and proliferation of primary CECs. A non-significant pro-survival effect was detected during regular cellular passage when CECs were pre-treated with Y-27632, an effect that became more evident during cryopreservation. Our study showed that the inclusion of Y-27632 was beneficial for the propagation of primary CECs expanded via the dual media approach, and was able to increase overall cell yield by between 1.96 to 3.36 fold.

The human cornea is a transparent, highly refractive structure of the eye and consists of five layers. The innermost single cell-layer is the corneal endothelium (CE), which plays a major role in the dynamic regulation of corneal hydration between its ‘leaky’ barrier and active fluid pumps^1^,^2^,^3^,^4^. In the eye, the cells of the corneal endothelial layer are locked in the G1-phase of cell cycle, mediated in part by tight cell-to-cell contacts^5^, as well as the presence of anti-proliferative factors such as transforming growth factor (TGF)-β2, found within the aqueous humor^6^. The non-proliferative state of the human CE *in vivo* prevents functional regeneration of damaged corneal endothelial cells (CECs). Hence, any loss of CECs results in the cellular enlargement of surviving adjacent CECs (polymegathism) to maintain functional integrity^1^. However, when extensive cell-loss of the CE layer occurs beyond a certain threshold such that the functional capacity of the remaining CECs becomes compromised, corneal decompensation will occur. This results in cornea edema that will eventually lead to corneal blindness^1^. Such phenomenon is often seen in patients afflicted by corneal endothelial dystrophies such as Fuchs' dystrophy^7^,^8^ or Congenital Hereditary Endothelial Dystrophy^9^,^10^.

Currently, restoring the vision of patients affected by these visually debilitating conditions can be achieved through surgical intervention. While a variety of surgical techniques have been developed^11^,^12^, including procedures that utilize all components of a donor cornea for treatments in multiple patients^13^, as well as the possibility of using alternative approaches instead of allograft corneal transplantation surgery in suitable patients^14^, conventional corneal transplants are still greatly limited by the availability of donor graft material^1^. This is a global problem that is further impeded by a myriad of factors, e.g. cultural restrictions to donation, that will in one way or another tap into the pool of donor corneas available^1^. Therefore, alternative approaches able to ease the surgical bottleneck are of great clinical interests. Currently, two potential alternatives are being explored. The first, ‘cell-injection therapy’, involves the direct injection of cultivated corneal endothelial cells into the anterior chamber of the eye^15^,^16^. The second, a broader approach under development by several groups around the world, involves the cell-tissue engineering of graft alternatives suitable for endothelial keratoplasty using cultivated cells grown or seeded on either a biological or synthetic scaffold carrier^17^,^18^,^19^. If successful, donor corneas, even those rejected for transplant due to low corneal endothelial cell counts^20^,^21^, can be set aside for cellular expansion for these alternative approaches. This however, requires the capacity to propagate human CECs in an *in vitro* setting.

Reports of human CEC-cultures were described as early as 1977 by Baum and colleagues^22^. At that time, difficulties were encountered in the propagation of CECs from donors over the age of 20, where confluence of culture took approximately 8 to 9 weeks to achieve and cellular morphology was heterogenic with evidence of CECs becoming multi-nucleated^22^. Since then, many reports of human CEC-culture have surfaced, some with more apparent success than others^1^. Many subtle changes have been made to improve human CEC-cultures over the years. For example, Shima and colleagues reported that using L-ascorbic acid 2-phosphate protected cultivated CECs against oxidative DNA damage and significantly increased the proliferation of human CECs through the up-regulation of hepatocyte growth factor (HGF) via a HGF/c-Met autocrine loop^23^,^24^. The selective activation of p120-catenin-Kaiso signaling to disrupt contact inhibition of CECs, bypassing canonical Wnt/β-catenin signaling, was able to prevent epithelial-to-mesenchymal-like transition (EMT) of expanded CECs^25^. The direct inhibition of TGF-β signaling using SB431542 and the use of bone morphogenetic protein-7 to inhibit TGF-β-mediated EMT has also been reported to prevent the fibroblastic transformation of expanded CECs^26^.

Small GTPase Rho and its downstream effectors, the Rho-associated coiled-coil protein kinases (ROCK), are associated with a range of cellular functions such as actin cytoskeleton organization, cell adhesion, G_1_-S progression of cell cycle, cytokinesis, and apoptosis^27^,^28^. The compound Y-27632, [(+)-(*R*)-*trans*-4-(1-aminoethyl)-*N*-(4-pyridyl)cyclohexanecarboxamide dihydrochloride], has been reported to be a selective ROCK inhibitor (ROCKi) of p160ROCK^29^, and has been shown to inhibit apoptosis, and increase cellular attachment and proliferation of both primate and human CECs^30^,^31^. However, Pipparelli and colleagues recently reported that inhibition of Rho-ROCK activity did not induce proliferation of human CECs, questioning the use of Y-27632 and its relevance in enhancing the proliferation rates of human CECs^32^.

We have recently described the propagation of human CECs using a dual media culture system^33^. The aim of this study was to systematically investigate the effects of ROCKi Y-27632 on the adherence, cell proliferation and cellular survival, for both regularly sub-cultured and cryopreserved cells, when applied during *in vitro* expansion of human CECs grown using the dual media approach. The incorporation of ROCKi into the isolation and propagation of CECs for three passages were also assessed.

## Results

### Characterization of human CECs and their exposure to Y-27632

**Figure 1:**
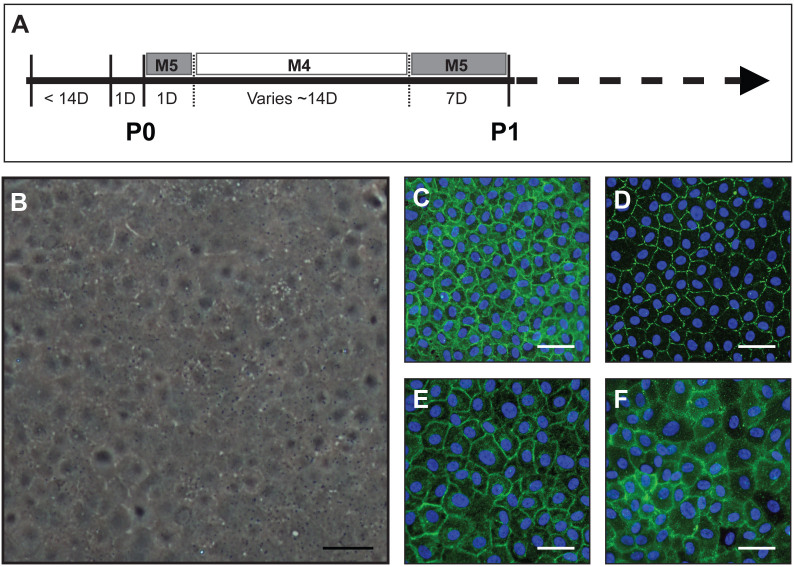
Culture and characterization of primary human CECs. (A) Schematic diagram depicting the expansion strategy of human CECs using a dual media approach, inter-switching between a M5-Endo medium for cellular stabilization/maintenance and a M4-F99 medium for cell proliferation. (B) Representative micrograph of a confluent homogeneous monolayer of P0 human CECs maintained for several days in M5-Endo medium following proliferative expansion in M4-F99 medium. Representative micrographic indirect immuno-fluorescent images of human CECs taken at the first passage which express (C) NA^+^/K^+^-ATPase, (D) ZO-1, (E) GPC-4 and (F) CD-200, indicative of the corneal endothelium. Scale Bars – B: 100 μm; C–F: 50 μm.

**Figure 2:**
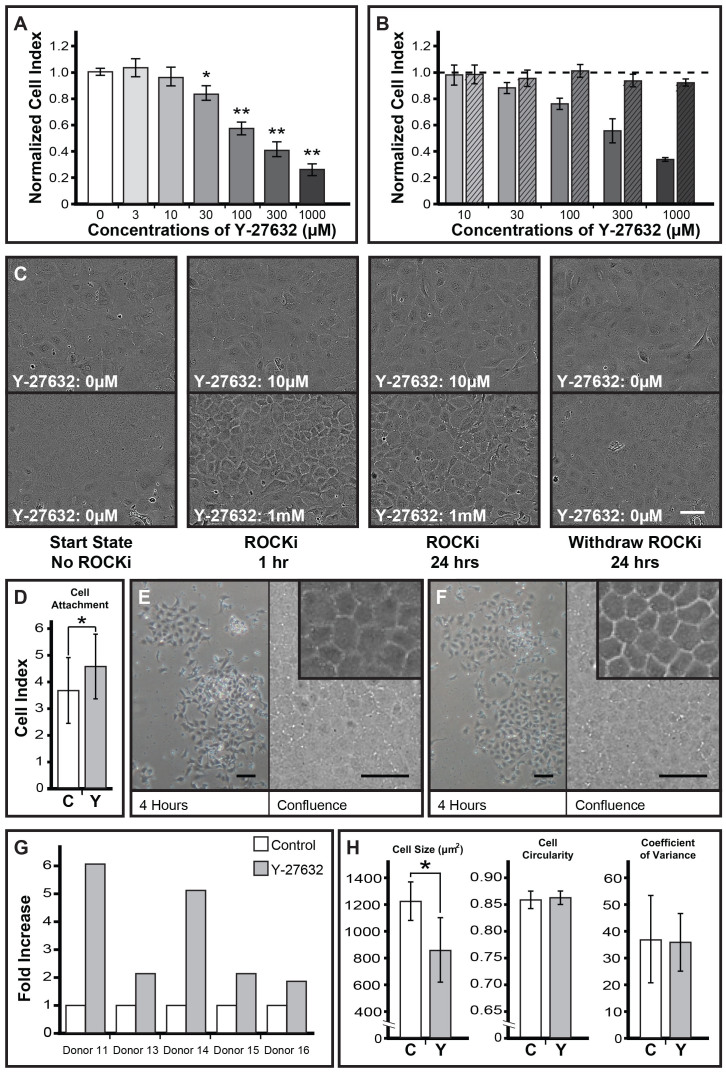
(A) Human CECs were seeded for 24 hours before exposure to various concentrations of Y-27632 (n = 7) including 3 µM, 10 µM, 30 µM*, 100 µM**, 300 µM**, and 1000 µM** for another 24 hours and analyzed using the xCELLigence system. (B) Normalized impedance readings of CECs treated for 24 hours, in various concentrations of Y-27632 (solid bars) and their respective recovery at Day 5 after the withdrawal of Y-27632 at Day 2 (striped bars). Cell index was normalized to 1 (dotted line) to untreated controls at 24 hours and at Day 5. (C) Representative time-lapse micrographs of CECs at start state, before the addition of Y-27632; 1 hour and 24 hours after the addition of Y-27632; and 24 hours after Y-27632 withdrawal. Either 10 µM (top panel) or 1 mM (bottom panel) of Y-27632 was used. (D) Impedance readings of CECs adherence at 4 hours, with or without the presence of 10µM of Y-27632 (n = 4) were measured using xCELLigence. Representative phase-contrast micrographs showing the morphology of CECs at 4 hours following cellular attachment (left panel) and at confluence (right panel), with insert showing a higher magnification of the confluent culture of (E) normal control P0 CECs and (F) their donor-matched counterparts which were exposed to 10 µM of Y-27632 throughout culture. (G) Overall cellular yield of isolated donor-matched P0 CECs growth to confluence with or without exposure to 10 µM of Y-27632. (H) Morphometric analysis (n = 3) – cell size*, cell circularity and coefficient of variance – of confluent donor-matched P0 human CECs grown with or without any exposure to 10 µM Y-27632 (*p < 0.05 and **p < 0.01) – For (D) and (H), ‘C’ denote control; ‘Y’ denote Y-27632 treated. Scale Bars: 100µm.

Primary human CECs were propagated using the dual media approach as depicted in the schematic (Fig. 1A). Isolated CECs grown using the dual media approach consistently generated a confluent homogenous monolayer of human CECs (Fig. 1B), and expressed characteristic pump-associated NA^+^/K^+^-ATPase (Fig. 1C), tight junction protein ZO-1 (Fig. 1D), heparin sulfate proteoglycan GPC-4 (Fig. 1E) and cell-membrane glycoprotein CD-200 (Fig. 1F), indicative of corneal endothelial cells^34^. To determine the optimal concentration of Y-27632 to be used in this study, we performed a cell impedance-based assay following the exposure of CECs to various concentrations of Y-27632. Concentrations of 30 μM and above showed a significant drop in the cellular impedance of CECs, following a 24-hours exposure, in a dose dependent manner (Fig. 2A). Interestingly, total withdrawal of ROCKi resulted in substantial recovery of impedance values across all ROCKi concentrations when assessed five days after withdrawal (Fig. 2B). Representative time-lapse micrographs showed a significant change in cell polarity of CECs when treated with 1 mM of Y-27632 compared to donor-matched control cells treated with 10 μM of Y-27632. However, upon withdrawal of Y-27632, cellular morphology of CECs reverted back to their initial state (Fig 2C). From this point onwards, a concentration of 10 μM of Y-27632 was used for the rest of this study.

### Y-27632 improves adherence and overall cell yield of human CECs

The adherence of human CECs onto FNC-coated cell-culture plates was assessed following the seeding of passaged cells (P1 or P2 cells) in M5-Endo medium with or without Y-27632. The cell attachment profiles of sub-cultured CECs were significantly better when Y-27632 was supplemented in M5-Endo medium during the attachment phase, as gauged by cell impedance readings taken from the xCELLigence system at the fourth hour following cell-seeding (Fig. 2D).

In a series of follow up experiments, we subjected freshly isolated human CECs from independent paired donor corneas, and divided them equally into cultures with or without the addition of Y-27632. As expected, cells in both conditions were seen to adhere well by the forth hour, and were able to form a homogenous monolayer with distinct polygonal/hexagonal morphology when they reached confluence (Fig. 2E and 2F). Interestingly, in these culture conditions, CECs grown in the presence of Y-27632 consistently yielded more CECs (between 1.86 fold to 6.07 fold more) for the 5 paired donors assessed (Fig. 2G). Morphometric analyses of cell size, cell circularity and coefficient of variance between donor matched-CECs samples grown with or without Y-27632 showed significant differences in the sizes of CECs measured (Fig. 2H). It was evident that CECs grown in the presence of Y-27632 were significantly smaller in size (880.21 ± 239.95 μm^2^) compared to donor-matched control cultures (1232.58 ± 143.35 μm^2^). No significant difference was observed for overall cell circularity and coefficient of variance between the two donor matched-CECs populations.

### Y-27632 enhances cell proliferation of human CECs in cells isolated from young donors

**Figure 3:**
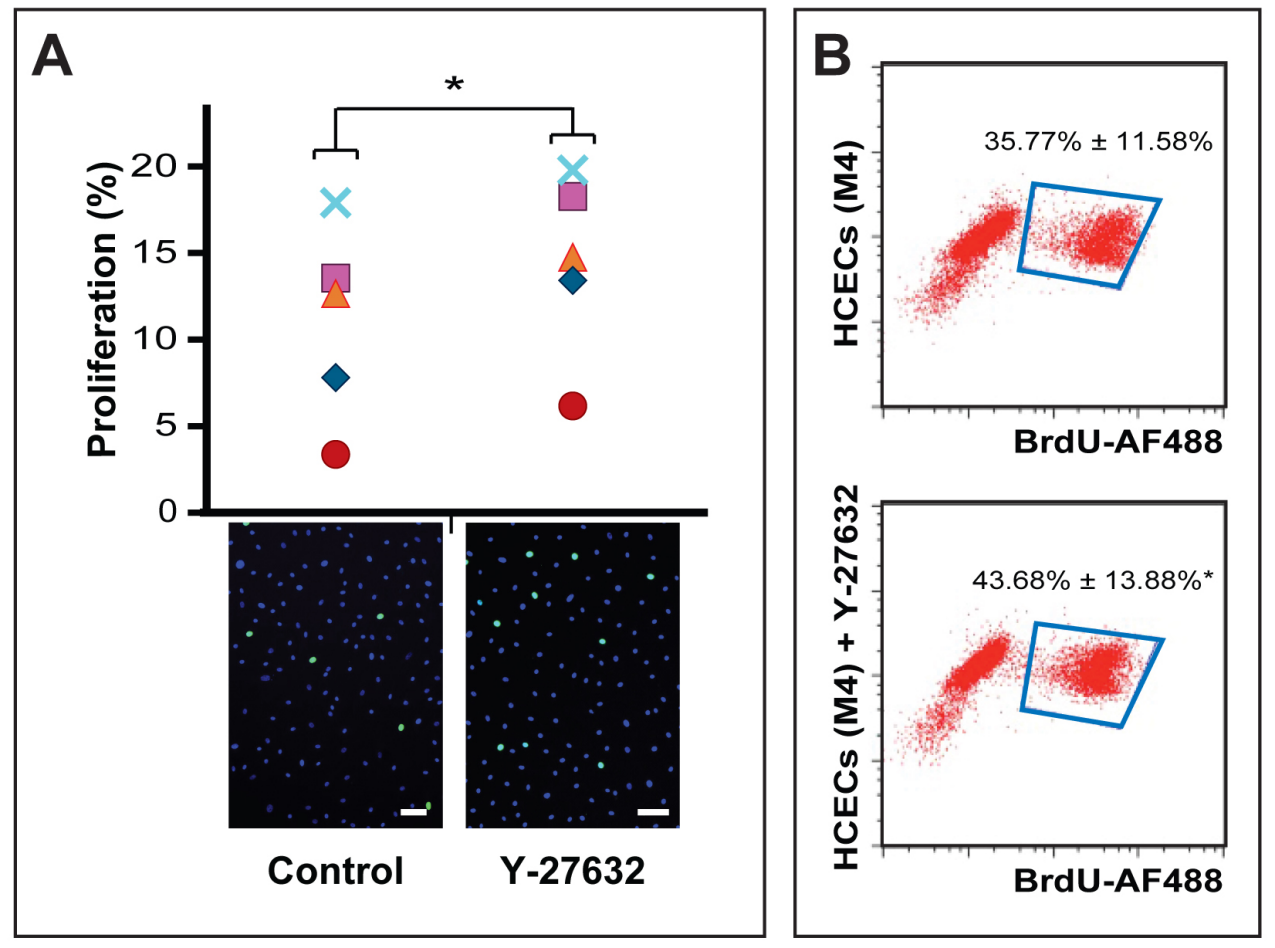
Exposure to Y-27632 increases the proliferation rates of primary human CECs. (A) Click-iT EdU assay was carried out to assess the cell proliferation of human CECs in M4-F99 medium with or without addition of 10 μM Y-27632 over a period of 24 hours (n = 5; *p < 0.05). (B) Representative flow cytometric dot plots of BrdU incorporation of human CECs grown in M4-F99 medium with or without exposure to 10 μM Y-27632 over a period of 48 hours (n = 4; *p < 0.05). Scale Bars: 100 μm.

The proliferation rates of human CECs were assessed following the switch from the initial M5-Endo medium into M4-F99 medium to initiate cellular proliferation. The proliferation rates were evaluated by 24-hours Click-iT EdU incorporation via immunofluorescence (Fig. 3A), and 48-hours BrdU incorporation using flow cytometric analysis (Fig. 3B). Results from Click-iT EdU incorporation assay showed that CECs expanded in medium containing Y-27632 contained more proliferative cells than their respective donor-matched controls grown in the same medium without Y-27632 (Fig. 3A). Although an increase in cell proliferation was detected across all donor samples, CECs from some donors showed a greater proliferative response when exposed to Y-27632 than others. Overall, the percentages of proliferative increase detected by EdU incorporation in the presence of Y-27632 were 0.96%, 1.54%, 2.62%, 4.91%, and 6.00% over a period of 24 hours (n = 5; Table 1). Results from flow analysis of 48-hours BrdU incorporation corroborated the above results, showing significant increase of CECs proliferation of 4.46%, 8.72% and 10.54% detected in the samples expanded in the presence of Y-27632 (n = 3; Table 1). Interestingly, when this study was repeated in human CECs isolated from older donors aged 60 and above, proliferative enhancement seen in younger donors were not detected in older samples cultured in Y-26732, and no statistical significance were detected (Supplementary Table 1).

### Y-27632 did not significantly improve the viability of human CECs during regular cellular passage or cryopreservation

**Figure 4:**
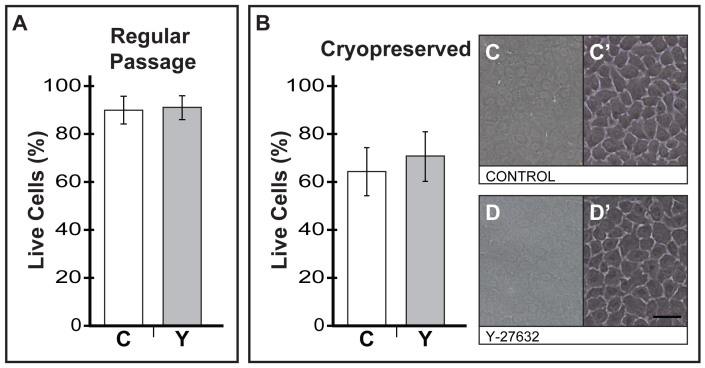
The use of Y-27632 for cell survival during regular cellular passage and recovery from cryo-preservation. (A) Confluent cultures of CECs maintained in M5-Endo medium, with or without a 1-hour pre-treatment of 10 μM Y-27632 were analyzed for overall percentage of live cells, as gauged by the Annexin V detection kit, following regular cellular passage - ‘C’ denote control; ‘Y’ denote Y-27632 treated (n = 6). (B) Overall percentage live cells were also assessed for both groups of cryo-preserved CECs that were either untreated or those that were exposed to 10 μM Y-27632 - ‘C’ denote control; ‘Y’ denote Y-27632 treated (n = 6). Representative micrographs of (C) cryo-preserved control or untreated CECs and (D) corresponding CECs that were exposed to 10 μM Y-27632 that were thawed out and seeded at physiological density of approximately 3,000 cells per mm^2^. (C′) and (D′) depicts CECs from corresponding micrographs (C) and (D) that were briefly treated with TrypLE Express for 5 seconds to better visualize the CECs. Scale Bars: 50 μm.

To study the effect of Y-27632 on the viability of human CECs during regular cellular passage, cells were pre-treated with Y-27632 at least 1 hour prior to cellular dissociation. The live-dead assay was immediately performed and analyzed by flow cytometer for total percentage live cells. During regular cellular passage, flow analysis (n = 6) showed that 89.52% ± 6.89% of the control CECs remained viable. Comparatively, donor-matched CECs pre-treated with Y-27632 showed 90.72% ± 6.35% in cell viability. These results suggested that the pre-treatment of Y-27632 did not confer any significant advantage to the survival of CECs during cellular passage (Fig. 4A). The live-dead assay was also conducted on cryopreserved CECs (n = 6). In the control group, approximately 63.96% ± 10.02% of CECs survived the process of cryopreservation. Donor-matched CECs that were exposed to Y-27632 were found to have survived better with 70.45% ± 10.35% of the cells that remained viable (Fig. 4B). However, the fractional increase in overall cell viability of approximately 6.49% ± 14.36% observed in cryopreserved CECs pre-treated with Y-27632 were not statistically significant. Nevertheless, when the cryopreserved groups of control CECs (Fig. 4C and 4C′) and Y-27632 pre-treated CECs (Fig. 4D and 4D′) were thawed out and seeded at a physiological density of 3,000 cells per mm^2^, both group of cells were successfully revived, and formed compact homogeneous polygonal/hexagonal cell layers following seven days of culture in M5-Endo medium.

### The use of Y-27632 is beneficial for the cellular expansion of human CECs

The final comparative study was performed using 4 pairs of donor-matched human CECs isolated and expanded to the second passage with or without Y-26732 added throughout the culture period. Result obtained showed that there were a marked increase in the overall cell-yield when Y-26732 is added, ranging from 1.96, 2.54, 2.67, and 3.36 folds (Table 2).

## Discussion

The isolation and propagation of primary human CECs has been described by various research groups using different approaches revolving around the use of a single basal medium, each supplemented with different additives^23^,^24^,^25^,^26^,^32^,^35^. However, most of these reports were not extensively characterized. We recently described the propagation of CECs via a dual media approach^33^. In that study, the isolated CECs were first established in M5-Endo medium overnight. Subsequently, the M5-Endo-stabilized CECs were propagated in a proliferative M4-F99 medium for their expansion until the CECs reached confluence, at which point the culture medium was switched back to M5-Endo medium. The incorporation of the M5-Endo medium, as depicted (Fig. 1A), was found to be effective in preventing potential endothelial-to-mesenchymal-like transition of the expanded CECs^33^. Indeed, the unique cellular morphology of human CECs (Fig. 1B), along with the expression of various markers indicative of the human corneal endothelium, such as the pump-associated NA^+^/K^+^-ATPase, tight junction protein ZO-1, heparin sulfate proteoglycan GPC-4, and cell-membrane glycoprotein CD-200, could be maintained (Fig. 1C–1F) by CECs expanded using this approach. Primary CECs propagated using the dual media approach have been characterized to the third passage, potentially generating between 1 × 10^7^ to 2.5 × 10^7^ CECs from a pair of donor corneas^33^,^36^. Although the dual media approach of propagating human CECs is relatively robust, the cultures of these primary cells are not without their limitations, and can be affected substantially by the procurement process as well as donor variations^37^,^38^. It should be noted that all the donor corneal pairs procured from Lions Eye Institute for Transplant and Research were processed within 14 days of preservation to minimize potential variation^38^. The successful establishment of a confluent culture of P0 human CECs is partly dependent on the final cellular yield of the isolated primary CECs, and their capacity to proliferate in M4-F99 medium, which can be potentially affected by inherent donor variation and other external factors^39^. Some of these factors include the relative health of the donor before death, the cause of death, as well as the duration from death to enucleation and preservation and the time from preservation to the establishment of culture^1^,^40^. Beyond these potential hindrances, we report herein that the dual media approach in expansion of human CECs can be further enhanced, in terms of absolute cellular yield, simply by the treatment of the cultured CECs with the well-described Rho-kinase inhibitor Y-27632^29^.

It is well established that the Rho-kinases are protein serine/threonine kinases that have pleiotropic functions, which include the regulation of cellular contraction, growth, migration, metabolism, polarity, as well as apoptosis^27^,^28^,^32^,^41^,^42^,^43^. In this study, three specific outcomes of ROCK inhibition using Y-27632 were assessed within the context of primary human CECs cultured using the dual media approach described^33^. These include cell-substrate adhesion; cellular proliferation, and general cell survival during regular cell passage, as well as cryopreservation. Even though it has been shown by various groups that a concentration of between 10 μM and 30 μM of Y-27632 can be used for the culture of human CECs^32^,^44^, and up to 100 μM of Y-27632 can be used without significant detrimental effect for the culture of primate CECs^30^, our results showed a dose-dependent drop in the adhesion strength when CECs were exposed to more than 30 μM of Y-27632 (Fig. 2A). This is not surprising, as ROCK is known to regulate cellular homeostasis^27^,^43^, and its inhibition has been associated with changes in actin stress fibers, as well as focal adhesion^45^, which in turn may affect adhesion strength. Similar occurrence has been observed in primary cell cultures of human trabecular meshwork (HTM) and Schlemm's canal (SC), where inhibition of Rho kinase by Y-27632 lead to reversible changes in cell shape, and actomyosin re-organization^45^. Indeed, time-lapse micrographs showed that the polarity of CECs was evidently affected by the higher dosage of Y-27632, and upon withdrawal, substantial recovery was observed, which coincided with the impedance readings of the cells (Fig. 2B). Taken together, these results suggested that concentrations above 30 μM of Y-27632 affect the attachment strength of CECs in a dose-dependent manner. It has been postulated that cellular relaxation and the loss of cell-substratum adhesion brought on by Rho kinase inhibition resulted from the decrease in myosin light-chain phosphorylation, in HTM and SC cells^45^, through the dephosphorylation of myosin light chain phosphatase complex^46^. However, it should be noted that the two studies used lower concentrations of Y-27632, 5 μM and 10 μM, as well as different cell types^45^,^46^. Hence, future studies on the phosphorylation state of myosin light-chain in primary human CECs measured in the presence of different concentration of Y-27632 will enable better understanding of the loss of adhesion strength observed in the present study.

The cellular adhesion of CECs to the extracellular matrix (ECM) substratum is an important parameter for the successful propagation of CECs. Although we and others have shown that CECs can be established directly onto uncoated cell-culture vessels, the seeding of CECs onto culture wares pre-coated with ECM such as FNC coating mixture, collagen type-I, collagen type-IV or laminin-5 significantly increases the attachment of CECs^35^,^38^,^47^,^48^. In this study, our results clearly showed that addition of Y-27632 during cell seeding further improved cell attachment of CECs on FNC-coated cell-culture plates by 38% ± 13% when impedance measurements were taken at the fourth hour (Fig. 2B). This finding is in line with previous reports that showed a significant increase in cellular adhesion of human CECs cultured with Y-27632^32^,^44^. Similar findings have been shown in the adhesion of primate CECs, where the inhibition of actin polymerization and up-regulation of vinculin were associated with the enhanced cell attachment in Y-27632 treated monkey CECs^49^. Rho and ROCK are known to regulate cell adhesion by enhancement of acto-myosin contractility^43^. Whilst it has been postulated that the RhoA-ROCK-PTEN signaling pathway may be integral to the reported findings^49^,^50^, further investigation is required to better understand the role of Y-27632 and the complex signaling mechanisms involved that resulted in the enhancement to the attachment of human CECs.

In a series of experiments, we found that freshly isolated donor-matched primary CECs, grown with or without the presence of Y-27632, adhered and proliferated well in the dual media culture system. Interestingly, CEC-populations exposed to Y-27632 generally reached confluence within a shorter period of time (unpublished observation), consistently yielded more CECs, and were remarkably more compact in cell-size (Fig. 2C–2F). The shorter time it took for CEC-cultures to reach confluence was most likely due to the increased proliferation rates as shown in CEC-cultures propagated in M4-F99 medium supplemented with Y-27632 (Fig. 3A and 3B). This observation is consistent with various reports showing increased cell proliferation of human CECs^31^,^44^, primate CECs^30^, and bovine CECs^51^ when Y-27632 was added to the culture medium. It has been demonstrated, at least in primate CECs, that PI-3-kinase signal cascade activated by Y-27632 resulted in the up-regulation of cyclin D and down-regulation of p27, which is central to the proliferation of CECs^31^. It should be noted that the use of Y-27632 to increase the proliferation of human CECs may only be appropriate for cultures of CECs established using younger donors, as the addition of Y-27632 were not found to be advantageous for cultures established using older donors (Supplementary Table 1). This is not surprising as similar observation was previously reported by Pipparelli and colleagues that Y-27632 has no effect on the proliferative capacities on human CECs in their study, which utilized corneas from donor aged 73 ± 3 years^32^. It has been widely reported that human CECs isolated from older donors were less proliferative than those established from younger donors, and this has been associated to significant increase of cyclin kinase inhibitors p16^INK4a^ and p21^WAF1/Cip1^, hence resulting in an age-dependent increase in negative regulation of cell cycle^52^.

There is a myriad of studies that have shown the involvement of ROCK signaling in the regulation of both pro-apoptotic signals, and anti-apoptotic or cell survival signals^27^,^43^. The dichotomy of the responses is believed to be stimulus and cell type-dependent^53^. For example, high intensity ROCK activity in cells placed under extended or elevated stressful conditions may contribute to the initiation of the apoptotic cascade, amplifying or even accelerating the apoptotic process; and under such situations, inhibition of ROCK signaling may produce a pro-survival outcome^41^. Indeed, it has been shown that the use of Y-27632 is effective in counteracting apoptosis in human embryonic stem cells, and it greatly improved the efficiency of clonal expansion of human embryonic stem cells^54^, as well as in the cryopreservation of human embryonic stem cells^55^,^56^. Cell survival of cultured primary primate CECs were also found to improve in the presence of Y-27632^30^. However, we did not detect any improvement to the overall cell survival of human CECs when passaged (Fig. 4A) or cryopreserved (Fig. 4B) in the presence of Y-27632 over donor-matched control cells. We speculate that these results may be in part due to the dual media culture system, specifically the M5-Endo medium that the human CECs were maintained in before they were passaged or cryopreserved. We have previously shown, by microarray analysis, that there were significant gene expression changes in the cultivated human CECs following the switch from M4-F99 medium to M5-Endo medium^33^. These changes may have conferred a protective effect on the cells that were maintained in M5-Endo medium. More importantly, human CECs expanded using the dual media approach can be cryopreserved, and subsequently revived with or without the use of Y-27632, to form a homogenous monolayer of polygonal CECs (Fig. 4C and Fig. 4D).

Taken together, results from this study as well as others^32^,^44^ - including studies using primate CECs^30^,^49^ indicate that the inhibition of ROCK signaling pathway using Y-27632 evidently influenced the propagation of primary CECs in a positive manner. It has since been demonstrated by Okumura and colleagues that the mechanistic actions of Y-27632 in promoting the proliferation of primary human CECs involve both cyclin D – a positive G1 regulator; as well as p27 – a negative G1 regulator, via PI 3-kinase signaling^31^. However, it should be noted that although Y-27632 has been touted as a selective inhibitor of ROCK^29^, it is known to inhibit other kinases such as citron kinase^43^,^57^. As such, further studies should aim at elucidating the exact interaction of Y-27632 within ROCK signaling, and if other ROCKi similar to Y-27632 such as Y-30141^58^ have similar effects when supplemented into the culture of primary CECs. Indeed, it has been shown that Y-39983, a molecule derived from Y-27632, but with higher potency in the inhibition of ROCK activity^59^ has shown some promise in increasing the proliferation of primate and human CECs^31^.

In conclusion, this study clearly showed that incorporating Y-27632 into the dual media culture approach of expanding primary human CECs is beneficial for the propagation of these primary cells, specifically CECs that were isolated from younger donors (below 40). The inclusion of Y-27632 into the respective media during the attachment phase (M5-Endo medium) and proliferative phase (M4-F99 medium) resulted in a 1.96 fold to 3.36 fold increase in overall cell yield by the second passage through the enhancement of cellular adherence, and increased cell proliferation (Table 2). We have projected, in a recent report, that approximately 30 tissue-engineered constructs can be generated from CECs isolated from a pair of donor corneas grown to the second passage using the dual media approach^60^. Hence, by adding Y-27632 into the dual media culture system, the increase in overall yield of CECs obtainable from a pair of donor cornea could potentially generate two to three times more tissue-engineered constructs. Hence, this can potentially increase the supply of transplantable graft materials, whilst substantially decreasing the costs of tissue-engineered endothelial constructs, making such intervention available to a wider group of patients^60^.

## Methods

### Materials

Ham's F12, Medium 199, Human Endothelial-SFM, fetal bovine serum (FBS), Dulbecco's Phosphate-Buffered Saline (PBS), Insulin/Transferrin/Selenium (ITS), TrypLE™ Express (TE), gentamicin, amphotericin B, Fix and Perm (Medium A), 5-ethynyl-29-deoxyuridine (EdU) incorporation Click-iT Alexa Fluor 488 cell proliferation assay kit, penicillin and streptomycin were purchased from Life Technologies (Carlsbad, CA, USA). Dimethyl Sulfoxide (DMSO), ROCKi Y-27632, trypan blue (0.4%), paraformaldehyde (PFA) and ascorbic acid were purchased from Sigma (St. Louis, MO, USA). Basic fibroblast growth factor was bought from R&D Systems (Minneapolis, MN, USA). FNC coating mixture was obtained from United States Biologicals (Swampscott, MA, USA). Collagenase A was purchased from Roche (Mannhein, Germany).

### Research-grade human corneoscleral tissues

All research-grade human cadaver corneal tissue procured for this study through Lions Eye Institute for Transplant and Research (Tampa, FL, USA) were obtained with written consent from the next of kin, and adhered to the principles outlined in the Declaration of Helsinki. A total of 33 pairs of donor corneal tissues ranged from 3 to 39 years old (Table 3) and four single donor conreal tissue between 60 and 66 years old (Table 4) with endothelial cell count of at least 2,000 cells per mm^2^ deemed unsuitable for transplantation were used for this study. Corneoscleral tissues were preserved in Optisol-GS (Bausch & Lomb, Rochester, NY, USA) at 4°C until they were processed, usually within 14 days of preservation.

### Cell Culture

Human CECs from paired donor corneas were isolated using a two-step ‘peel and digest’ method^38^, and propagated using the dual media approach as described^33^, with some modifications to evaluate the effect of Y-27632. Briefly, isolated CECs were first established in cornea endothelial maintenance/stabilization medium (M5-Endo; Human Endothelial-SFM supplemented with 5% FBS) overnight. Subsequently, CECs were cultured in corneal endothelial proliferation medium (M4-F99; Ham's F12/M199, 5% FBS, 20 μg/ml ascorbic acid, 1× ITS, and 10 ng/ml bFGF) to promote the proliferation of human CECs^33^. Once CECs become 80%–90% confluent, M5-Endo was re-introduced to the culture for at least two days before being sub-cultured via TE dissociation. Dissociated CECs were plated at a seeding density of at least 1 × 10^4^ cells per cm^2^ on FNC-coated surfaces for cellular expansion^36^. All cultures were incubated in a humidified atmosphere at 37°C and 5% CO_2_.

### Immunocytochemistry

Confluent P2 human CECs were passaged and plated at a density of at least 2,000 cells per mm^2^ on FNC-coated glass coverslips and maintained for approximately seven days in M5-Endo before fixation with the appropriate fixative (Table 5). Samples were rinsed and blocked in 5% normal goat serum in PBS for 30 min at room temperature. Subsequently, samples were incubated with the primary antibodies at room temperature for 1 hour or at 4°C overnight. Primary antibodies used in this study are as listed in Table 5. The samples were then washed twice with PBS, 5 min each, and labeled with AlexaFluor 488 conjugated goat anti-mouse IgG secondary antibody (2.5 μg/ml, Life Technology) for 1 hour at room temperature in the dark. After two brief PBS washes, samples were mounted in Vectashield containing DAPI (Vector Laboratories, Burlingame, CA, USA), and visualized under a fluorescence microscope.

### Characterization of Y-27632 concentrations and cell adhesion assay

To determine the optimal concentration of Y-27632 for cultivated human CECs, expanded P2 or P3 human CECs were seeded at a density of 2.5 × 10^4^ cells per cm^2^ in each well of an E-Plate 96 (ACEA Biosciences, San Diego, CA, USA), and cultured for 24 hours in M5-Endo. On the second day, CECs were exposed to M5-Endo containing different concentrations (3, 10, 30, 100, 300, and 1000 μM) of Y-27632 for another 24 hours. Subsequently, Y-27632 was withdrawn and CECs were cultured for another five days in fresh M5-Endo medium. The electrical impedance readings of the cells were recorded using the xCELLigence real time cell analyzer (ACEA Biosciences) throughout the experiment. To document the morphology of CECs exposed to 10 μM and 1000 μM of Y-27632, time-lapse images were acquired using an IncuCyte ZOOM live cell imager (Essen BioScience, Ann Arbor, MI, USA). Viewing areas were randomly selected and images were captured at 30-minute intervals. To assess the effect of Y-27632 on the adherence of human CECs, P2 or P3 cells were dissociated using TE and seeded into E-Plate 96 as described above, with or without the presence of 10 μM of Y-27632 in M5-Endo. The seeded human CECs were equilibrated for 30 minutes in the incubator before the impedance reading depicting the adherence profile of the CECs were captured for 24 hours.

### Cell proliferation assay

Click-iT EdU Assay: Proliferation rates of human CECs propagated with or without Y-27632 were assessed using the EdU incorporation Click-iT cell proliferation assay as per manufacturer's instructions. Briefly, cultured CECs passaged using TE were seeded onto FNC-coated glass slides at a density of 5 × 10^3^ cells per cm^2^ and maintained in M5-Endo for 24 hours. On the second day, the medium was switched to either M4-F99 or M4-F99 containing 10 μM of Y-27632, and cells were cultured for another 24 hours. On the third day, cells were incubated in M4-F99 containing 10 μM of EdU for another 24 hours. Subsequently, samples were rinsed once with PBS followed by fixation with freshly prepared 4% PFA for 15 mins, on ice. Samples were rinsed twice with PBS, followed by incubation in 0.1% Triton X-100 in 3% BSA in PBS for 20 minutes at room temperature for blocking and permeabilization. Incorporated EdU was detected by fluorescent-azide coupling Click-iT reaction where samples were incubated for 30 minutes in a reaction mixture containing 1× Click-iT EdU reaction buffer, CuSO_4_, and azide-conjugated Alexa Fluor 488 dye, in the dark. Samples were rinsed with PBS and mounted in Vectashield containing DAPI. A Zeiss Axioplan 2 fluorescence microscope (Carl Zeiss, Germany) was used to examine the labeled proliferative cells. At least 250 nuclei were analyzed for each experimental set.

BrdU Incorporation Assay: The effect of Y-27632 on proliferation of human CECs was also investigated via a BrdU incorporation assay, where flow cytometric analyses were performed using the MACSQuant Analyzer (MACS Miltenyi Biotec, Bergisch Gladbach, Germany). Here, human CECs were plated at a density of 5 × 10^3^ cells per cm^2^ on FNC-coated cell-culture plates overnight in M5-Endo. On the second day, the medium was switched to either M4-F99 or M4-F99 containing 10 μM of Y-27632, together with BrdU for pulse-labeling, and cells were grown for another 48 hours. For the analysis of BrdU labeled cells, samples were processed as per manufacturer's instructions, described in the FlowCellect Bivariate Cell Cycle Analysis Kit for DNA replication. Briefly, labeled cell samples were dissociated with TE, spun down, and the supernatant removed. Samples were then fixed using the fixation buffer for 20 minutes on ice, and permeabilized with the permeabilization buffer for 5 minutes on ice before being treated with 300 μg/mL DNase I (Sigma) for 1 hour at 37°C. Subsequently, the cells were incubated with Anti-BrdU Alexa Fluor 488 for 1 hour in the dark on ice as indicated. Labeled cells were analyzed for BrdU incorporation using the MACSQuant Analyzer and a minimum of 10,000 events were captured.

### Cell survival assay

To assess the effect of Y-27632 on human CECs during regular cellular passage, confluent CEC-cultures were pre-treated with Y-27632 one hour before TE dissociation. Corresponding donor-matched control CEC-cultures were not exposed to Y-27632. Viable and dead CECs were distinguished using Fluorescein isothiocyanate (FITC) Annexin V apoptosis detection kit with Propidium Iodide (BioLegend, San Diego, CA, USA) following the manufacturer's instructions, which enables discrimination between viable cells (Annexin V-FITC^−ve^/PI^−ve^); early apoptotic cells (Annexin V-FITC^+ve^/PI^−ve^); and late apoptotic cells (Annexin V-FITC^+ve^/PI^+ve^). Briefly, harvested single cells, with or without pre-treatment of Y-27632, were re-suspended and incubated in solution containing Annexin V-FITC and PI, in the dark for 15 minutes, before being analyzed using a FACS Verse flow cytometer (Becton Dickinson, East Rutherford, NJ, USA).

To evaluate the effect of Y-27632 on cryopreservation of human CECs, confluent cells cultured with and without the 1 hour pre-treatment of Y-27632 were dissociated via TE, centrifuged and resuspended in freezing medium consisting of 10% DMSO in M5-Endo. A total of 5 × 10^5^ cells were dispensed into each cryovial (Nalgene, Thermo Fisher Scientific, Waltham, MA, USA), and immediately placed in a freezing container (Nalgene) containing pre-cooled isopropyl alcohol (Merck Millipore, Billerica, MA, USA) and transferred to a −80°C freezer overnight. The following day, cryovials were transferred and stored in liquid nitrogen. After at least one week of storage in liquid nitrogen, cryovials were retrieved and thawed in a 37°C water bath. After the removal of the freezing medium via centrifugation, cell pellets were resuspended and divided, where half was placed in a solution containing Annexin V-FITC and PI and analysed via flow cytometry as described above. The remaining CECs were seeded at a density of 3,000 cells per mm^2^ and maintained in M5-Endo for at least seven days.

### Statistics

All numeric data obtained were expressed as mean ± standard deviation (SD). All statistical analyses were performed using SPSS Statistics 22.0 (IBM, Chicago, IL, USA). The comparison of cell impedence of human CECs exposed to various concentrations of Y-27632 was performed using one-way ANOVA followed by post-hoc Bonferroni test for multiple comparisons (Figure 2A). All other statistical comparisons between non-treated human CECs (control) against donor-matched Y-27632-treated human CECs were evaluated using paired samples *t*-tests. All results with a *p*-value of less than 0.05 were deemed to be statistically significant.

## Author Contributions

G.P., D.T.T. and J.S.M. conceived and designed the study; G.P., K.A., B.L.G., H.P.A., and X.Y.S. carried out experiments and contributed to different aspects of data analysis. K.A. coordinated the study and together with G.P. and J.S.M. wrote the manuscript.

## Supplementary Material

Supplementary Information Supplementary Table 1

## Figures and Tables

**Table 1:** Proliferation rates of human CECs with and without Y-27632

Donor	M4-F99 Medium (Control)	M4-F99 Medium (Y-27632)	Overall increase
Click-iT EdU incorporation for 24 hours			
08	3.43%	6.05%	2.62%
09	18.75%	19.71%	0.96%
10	13.27%	18.18%	4.91%
17	7.69%	13.69%	6.00%
23	12.74%	14.29%	1.54%
BrdU incorporation for 48 hours			
19	35.42%	45.96%	10.54%
20	24.37%	28.83%	4.46%
21	47.53%	56.25%	8.72%

In the presence of Y-27632, an increase of approximately 0.96% to 6.00% of proliferating primary human CECs were observed when assessed after 24 hours click-iT EdU incorporation, and an increase of approximately 4.46% to 10.54% were detected when assessed after 48 hours BrdU incorporation.

**Table 2:** Human CECs expansion profile with and without the presence of Y-27632

Donor	OD Control @ P2	OS ROCKi @ P2	Fold Increase
17	260,000	510,000	1.96
22	1,845,000	4,680,000	2.54
23	617,000	2,070,000	3.36
24	708,000	1,890,000	2.67

Expansion profiles of primary human CECs propagated to the second passage were compared. Estimated cell-yield of CECs expanded using the dual media approach as control (OD) was compared to donor-matched CECs grown in the dual media approach supplemented with Y-27632 throughout the study (OS). An increase of at least 1.96 fold and up to 3.36 fold was observed in studies conducted using CECs isolated from 4 independent donors.

**Table 3:** Donor information

						F				T	
Serial Number	Age	Sex	Days to Culture	Cell Count (OS/OD)	Cause of Death	1	2	3	4	3	4
01	18	M	8	2907/3040	Motor Vehicle Accident	•	•				
02	39	F	9	3125/2882	Anoxia		•				
03	32	M	7	3021/2427	Sepsis	•	•				
04	27	F	6	2203/2037	Sepsis		•				
05	17	F	6	3058/3185	Motor Vehicle Accident	•	•				
06	20	M	14	3584/3704	Myocardial Infarction		•				
07	11	F	7	3534/3584	Anoxia	•	•				
08	35	F	5	2899/2941	Overdose		•	•		•	
09	31	F	9	2591/2611	Overdose		•	•		•	
10	19	F	7	2681/2882	Acute Cardiac Crisis		•	•		•	
11	3	F	5	3650/3690	Drowning	•	•				
12	15	F	6	2778/2770	Anoxia	•	•				
13	22	M	11	2604/2950	Possible Overdose	•	•				
14	18	F	14	3922/3953	Myocardial Infarction	•	•				
15	19	M	10	2890/2703	Leukemia	•	•				
16	19	M	10	3175/3067	Motor Vehicle Accident	•	•				
17	28	F	7	2778/2857	Myocardial Infarction			•		•	•
18	21	M	8	3322/3030	Motor Vehicle Accident		•				
19	20	M	11	3876/3663	Anoxia			•			
20	35	M	10	3115/2857	Overdose			•			
21	38	F	5	3356/3425	Acute Cardiac Crisis			•			
22	19	M	10	2717/2577	End Stage Lung Disease						•
23	21	M	6	2890/2974	Leukemia			•		•	•
24	35	F	10	2268/2331	Sepsis						•
25	9	M	10	3247/3096	Anoxia	•			•		
26	3	M	9	3968/4082	Drowning	•			•		
27	23	M	5	3311/3413	Motor Vehicle Accident				•		
28	20	F	9	3472/3300	Overdose				•		
29	14	F	5	3115/3205	Cerebral Edema				•		
30	9	M	10	3846/3774	Total Heart Block				•		
31	35	M	8	2632/2770	Pneumonia		•				
32	33	M	11	2404/2584	Gun Shot Wound		•				
33	31	M	5	2747/2525	Motor Vehicle Accident		•				

Donor age ranged from 3 year-old to 39 year-old with a median age of 20 year old. Days taken from death of donor to the initiation of corneal endothelial cell culture ranged from 5 days to 14 days with a median of 8 days. Corneal endothelial cell counts of corneas procured were over 2,000 per mm^2^. Data points generated in this study from cultures of human CECs that contributed to the respective figures and tables were denoted by ‘•’. OS: *oculus sinister*; OD: *oculus dexter*; F: Figure; T: Table.

**Table 4:** Corneas from four older donors aged 60 and above were also procured for this study. Days taken from death of donor to the initiation of corneal endothelial cell culture ranged from 4 days to 8 days. Corneal endothelial cell counts of corneas procured were over 2,000 per mm^2^

Serial Number	Age	Sex	Days to Culture	Cell Count (OS or OD)	Cause of Death
34	66	M	7	2597	End Stage Renal Disease
35	65	F	6	2703	Cerebrovascular Accident
36	60	M	4	2494	Myocardial Infarction
37	65	M	8	2577	Cerebrovascular Accident

Single corneas from four older donors aged 60 and above were also procured for this study. Days taken from death of donor to the initiation of corneal endothelial cell culture ranged from 4 days to 8 days. Corneal endothelial cell counts of corneas procured were over 2,000 per mm^2^. Data points generated from cultures of human CECs in this study from cultures of human CECs that contributed to the respective figures and tables were denoted by ‘**·**’. OS: *oculus sinister*; OD: *oculus dexter*; F: Figure; T: Table.

**Table 5:** Information of primary antibody used in this study

Antibody (Clone)	Company (Catalog Number)	Fixative	Concentration
Na^+^/K^+^-ATPase (0.T.1)	Santa Cruz (sc-71638)	100% Ethanol 5 minutes, 4°C	5 μg/mL
ZO-1 (1/ZO-1)	BD Pharmingen (610966)	100% Ethanol 5 minutes, 4°C	5 μg/mL
Glypican-4 (MAFB4044)	Creative Biomart (CAB-244MH)	Fix and Perm (Medium A) 15 minutes, room temp.	10 μg/mL
CD-200 (MRC OX-104)	BD Pharmingen (552023)	4% freshly made PFA 10 minutes, 4°C	10 μg/mL

The various primary antibodies used in this study are listed here by their clone name, the company of which the primary antibody was purchased from, with their respective catalog numbers. The different methods of fixation and concentration used were optimized for the staining of primary human CECs.
