# Supplementary material for: The effects of Rho-associated kinase inhibitor Y-27632 on primary human corneal endothelial cells propagated using a dual media approach
Source: Sci Rep. 2015 Mar 16;5:9167. doi: 10.1038/srep09167 (PMC4387913; doi:10.1038/srep09167)
Supplement: Supplementary Information — Supplementary Table 1 [file srep09167-s1.pdf]

Manuscript Title:

The effects of Rho-associated kinase inhibitor Y-27632 on primary human corneal endothelial cells propagated using a dual media approach

Gary S.L. Peh,[1,2] *Khadijah Adnan,*[1] *Benjamin L. George,*[1] *Heng-Pei Ang,*[1] *Xin-Yi Seah,*[1] *Donald T. Tan,*[1,3,5] *Jodhbir S. Mehta*[1,2,3,4].

[1] Tissue Engineering and Stem Cell Group, Singapore Eye Research Institute, Singapore.
[2] Duke-NUS Graduate Medical School, Singapore.
[3] Singapore National Eye Centre, Singapore.
[4] School of Material Science and Engineering, Nanyang Technological University, Singapore.
[5] Yong Loo Lin School of Medicine, National University of Singapore, Singapore.

Supplementary Table 1. Proliferation rates of human CECs isolated from older donors (aged 60 and above) with and without Y-27632

Donor	Age	M4-F99 Medium (Control)	M4-F99 Medium (Y-27632)
34	66	22.3%	16.2%
35	65	25.6%	26.8%
36	60	9.3%	6.2%
37	66	1.2%	1.8%

For human CECs isolated from older donors aged 60 years and above, Click-iT EdU incorporation assay showed no statistical significance in the proliferation of CECs in the presence of Y-27632 when compared to non-treated controls.
